# Supplementary material for: Selective amide bond formation in redox-active coacervate protocells
Source: Nat Commun. 2023 Dec 21;14:8492. doi: 10.1038/s41467-023-44284-x (PMC10739716; doi:10.1038/s41467-023-44284-x)
Supplement: Supplementary file 1 — Supplementary information [file 41467_2023_44284_MOESM1_ESM.pdf]

Supplementary information

Selective amide bond formation in redox-active coacervate protocells

Jiahua Wang,[a],[b] Manzar Abbas,[a] Junyou Wang*[c] and Evan Spruijt* [a]

[a] Institute for Molecules and Materials, Radboud University, Heyendaalseweg 135, 6525 AJ, Nijmegen, the Netherlands

[b] Department of Radiology, Shanghai Jiao Tong University School of Medicine Affiliated Sixth People's Hospital, Shanghai, 200233, China.

[c] State Key Laboratory of Chemical Engineering, East China University of Science and Technology, Shanghai, 200237, China

* Correspondence: e.spruijt@science.ru.nl, junyouwang@ecust.edu.cn

Table of contents

Table of contents 2

1. Synthesis and characterization of compounds 3
   1.1. Synthesis of trimethylated oligolysine and poly-L-lysine 3
   1.2. Synthesis of sodium 2-acetamidoethanethioate Ac-Gly-S-Na$^+$ 3
2. Formation and characterization of coacervates 4
   2.1. Coacervate fluidity 4
   2.2. Coacervate appearance with different polycations 5
   2.3. Coacervate appearance after ligation reaction 5
   2.4. Partitioning of client molecules 5
   2.5. Critical salt concentration (CSC) 7
   2.6. Redox reactions inside coacervates 7
3. Peptide bond formation reactions 8
   3.1. Control reactions without ferricyanide 8
   3.2. Reference ligation reaction in supernatant phase 9
   3.3. Control reactions with coacervates in borate buffer 9
   3.4. Coacervate-mediated ligation reactions with individual amino acids 10
   3.5. Coacervate-mediated ligation reactions with mixtures of amino acids 13
4. Fiber self-assembly inside coacervates 19
5. Reaction with aminonitriles 21
6. Supplementary references 22

1. Synthesis and characterization of compounds

1.1. Synthesis of trimethylated oligolysine and poly-L-lysine

Trimethylated oligolysines $(Lys(Me)_3)_n$ and poly-L-lysine ($pLys(Me)_3$) were prepared from $(Lys)_n$ and pLys, according to literature methods.[1] Briefly, 2 mL dimethyl sulfate (DMS) was added to 50 mg $(Lys)_n$, in 20 mL H_2O and 6 mL ethanol. The pH was adjusted to 9.5 and maintained by the addition of 1 M NaOH. The reaction was considered complete when the pH remained nearly constant. The $pLys(Me)_3$ was further purified by dialysis against 2 times 1 L of a 2 M NaCl aqueous solution and subsequently, 3 times 1 L of water in a dialysis membrane (MWCO, 3500). After that, the residue was freeze-dried to yield a white powder.

1.2. Synthesis of sodium 2-acetamidoethanethioate Ac-Gly-S-Na⁺

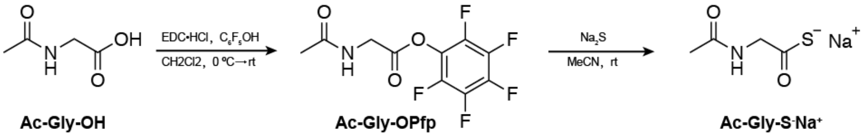

Ac-Gly-S⁻Na⁺ was synthesized according to literature methods (Supplementary Fig. 1-3).[2-3] Specifically, *N*-(3-Dimethylaminopropyl)-*N′*-ethylcarbodiimide hydrochloride (EDC·HCl) (3.45 g, 18.00 mmol) was added to a stirring solution of *N*-acetylglycine **Ac-Gly-OH** (943 mg, 6.00 mmol) and pentafluorophenol (1.22 g, 6.60 mmol) in CH_2Cl_2 (25 mL) at 0 °C. The reaction mixture was allowed to warm to room temperature and stirred for 16 h. The resultant homogenous solution was diluted with CH_2Cl_2 (20 mL) and washed with water (2 x 20 mL), $NaHCO_3$ (sat., 2 x 20 mL) and brine (20 mL). The organic layer was dried over Na_2SO_4, filtered, and concentrated in vacuo to give *N*-acetylglycine pentafluorophenyl ester **Ac-Gly-OPfp**, which was used immediately without further purification. The crude **Ac-Gly-OPfp** was resuspended in anhydrous acetonitrile (15 mL) and stirred vigorously with anhydrous sodium sulfide (1.1 equiv) under argon atmosphere for 6 h at room temperature. The resultant precipitate was isolated by centrifugation and washed with diethyl ether (3× 10mL) and lyophilized to yield **Ac-Gly-S⁻Na⁺** as a white solid (350 mg). ^1H NMR (400 MHz, D_2O): d 4.08 (s, 2H, (C2)–H_2), 1.97 (s, 3H, ($COCH_3$). HRMS-ESI $[M–H]^-$ calcd. for $[C_4H_7NO_2S–H]^-$: 132.0119; observed: 132.0117.

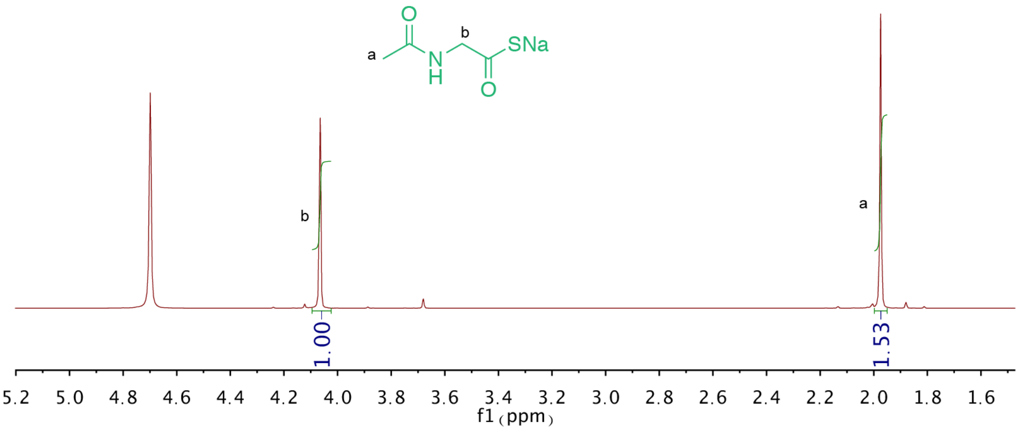

Supplementary Figure 1: Synthesis of sodium 2-acetamidoethanethioate. ^{1}H-NMR spectrum of Ac-Gly-$S^{-}Na^{+}$ (400 MHz, D_2O).

2. Formation and characterization of coacervates

2.1. Coacervate fluidity

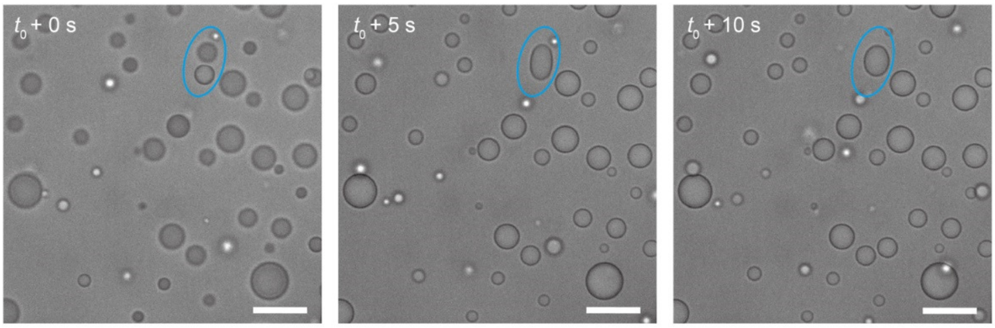

Supplementary Figure 2: Fusion of coacervates. Fusion of $Fe(CN_6)^{3-}$/pLys coacervate droplets (2 mM $Fe(CN_6)^{3-}$, 5 mM pLys monomer basis). The experiment was independently repeated three times with similar results. Scale bar, 10 µm.

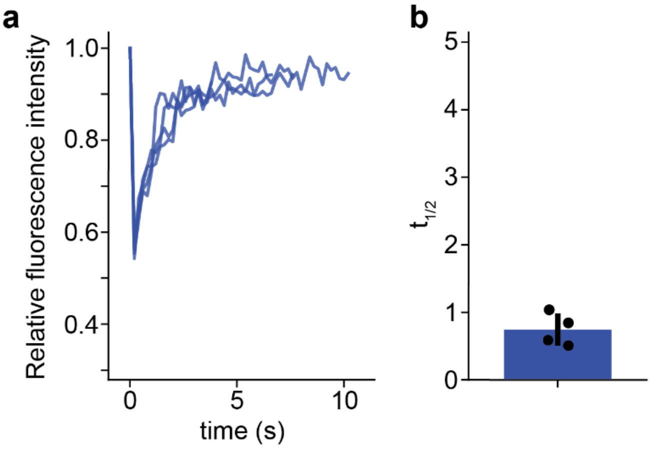

Supplementary Figure 3: FRAP. (a) Fluorescence recovery after photobleaching of Alexa 647-labelled K_{30} in $Fe(CN_6)^{3-}$/$(Lys)_{30}$ coacervates (5 mM $Fe(CN_6)^{3--}$, 5 mM pLys monomer basis, with 10% labelled K_{30}). (b) The corresponding half-time of recovery ($t_{1/2}$) of ferricyanide/$(Lys)_{30}$ coacervates in seconds.

2.2. *Coacervate appearance with different polycations*

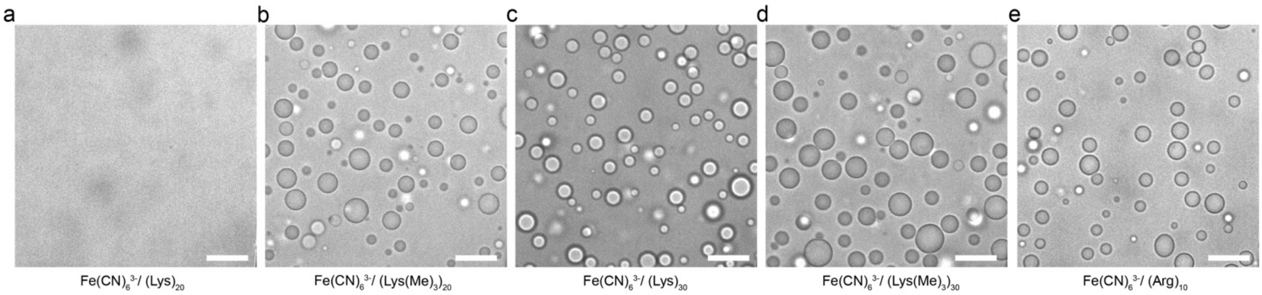

Supplementary Figure 4: Ferricyanide-based coacervates with varying polycations. Optical microscope images of $Fe(CN_6)^{3-}/(Lys)_{20}$ (a), $Fe(CN_6)^{3-}/(Lys(Me)_3)_{20}$ (b) (corresponding bright filed image for Fig. 3 in the main article), $Fe(CN_6)^{3-}/(Lys)_{30}$ (c), $Fe(CN_6)^{3-}/(Lys(Me)_3)_{30}$ (d) (corresponding bright filed image for Fig. 3 in the main article), and $Fe(CN_6)^{3-}/(Arg)_{10}$ (e) coacervates (corresponding bright filed image for Fig. 4 in the main article). The experiments in **a-e** were independently repeated three times with similar results. Scale bar 10 µm.

2.3. *Coacervate appearance after ligation reaction*

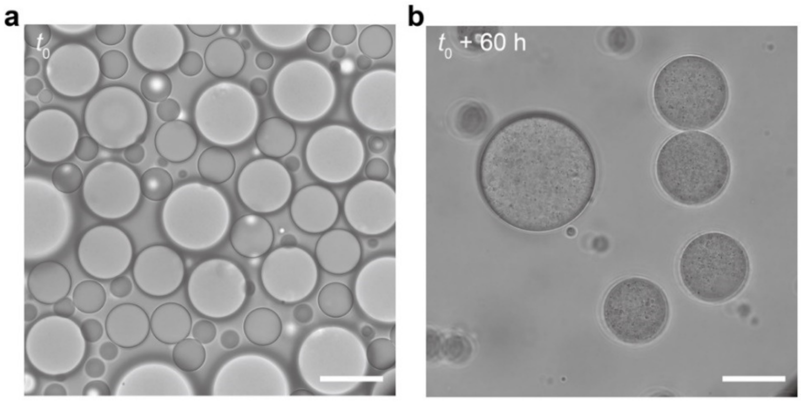

Supplementary Figure 5: Coacervate appearance after ligation reaction. Optical microscope images of $Fe(CN_6)^{3-}$/peptide coacervates, before (a) and after (b) peptide ligation reaction, as described in Figure 3. The dark spots inside the coacervates in (b) are colloidal sulfur clusters. The experiment was repeated three times with similar results. Scale bar, 20 µm.

2.4. *Partitioning of client molecules*

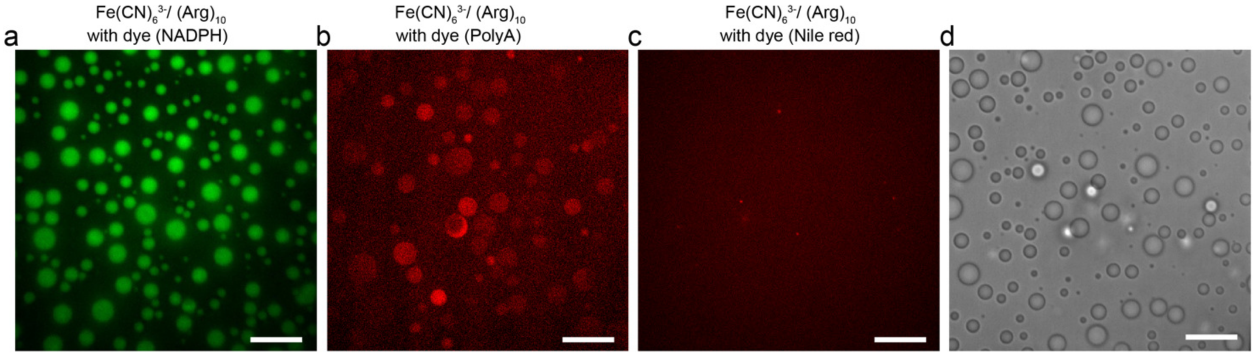

Supplementary Figure 6: Partitioning of dyes. Fluorescence images of $Fe(CN_6)^{3-}/(Arg)_{10}$ coacervate droplets selectively uptake of NADPH (a), Poly-A_{15} (Cy5-A_{15}) (b), and Nile red (c). (d) Corresponding bright field images of (c). The experiments in **a-d** were independently repeated three times with similar results. Scale bar, 10 µm.

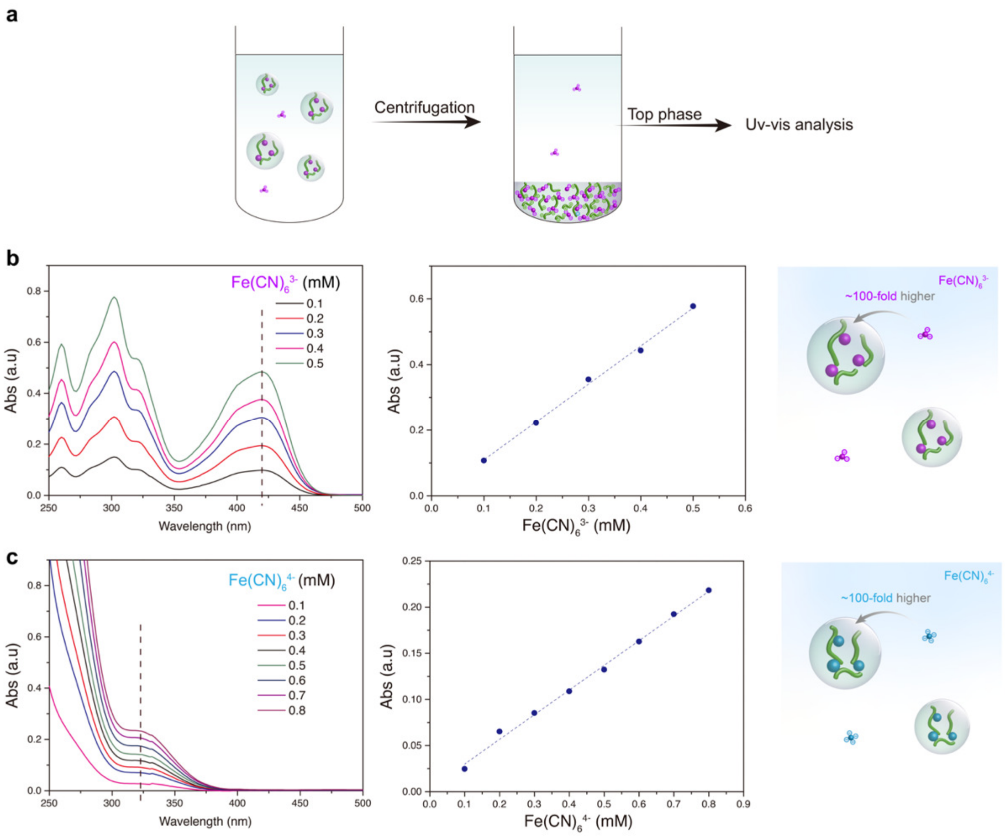

Supplementary Figure 7: Partitioning of ferricyanide and ferrocyanide. (a) Scheme of the method to determine the $Fe(CN_6)^{3-}$ concentrations inside and outside the droplet phase. (b, c) UV-vis spectra of the $Fe(CN_6)^{3-}$ or $Fe(CN_6)^{4-}$ concentration in top phase at different concentrations, and the corresponding standard curve of the $Fe(CN_6)^{3-}/Fe(CN_6)^{4-}$ absorbance. We calculated the $Fe(CN_6)^{3-}$ and $Fe(CN_6)^{4-}$ concentrations of the top solution from the standard curve, and then the $Fe(CN_6)^{3-}$e and $Fe(CN_6)^{4-}$ concentrations inside the coacervate phase can be known.

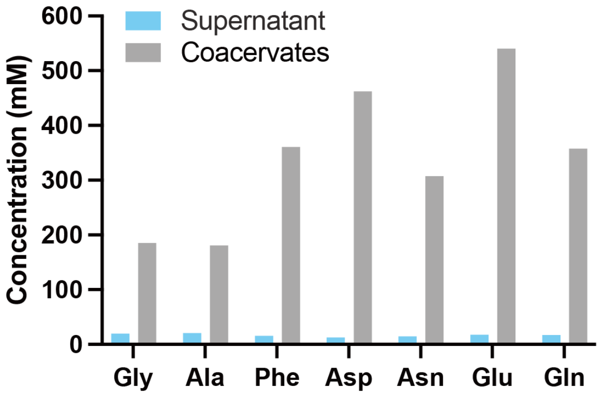

Supplementary Figure 8: Local concentrations of amino acids. Concentrations of amino acids in the coacervate phase and supernatant.

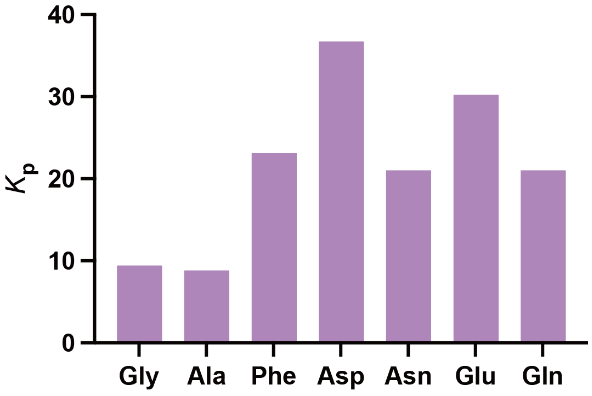

Supplementary Figure 9: Partitioning of amino acids. The partitioning coefficients of amino acids in coacervates.

2.5. *Critical salt concentration (CSC)*

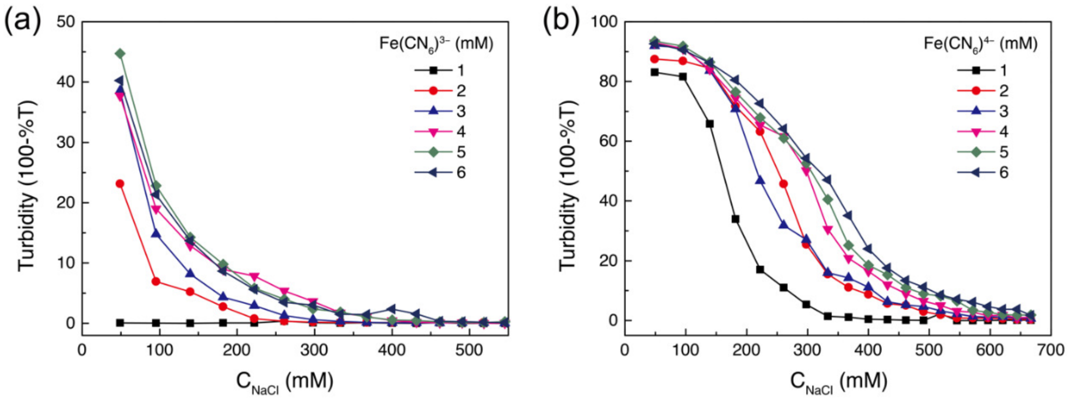

Supplementary Figure 10: Critical salt concentration. Turbidity of $Fe(CN)_6^{3-}$/$Fe(CN)_6^{4-}$-pLys mixtures as a function of concentration of added NaCl (a and b). The mixtures contained a fixed concentration of 50 mM Tris buffer, 5 mM pLys (15-30 kDa, monomer units). The mixtures containing $Fe(CN_6)^{3-}$ were titrated with NaCl 0.5 M, while the mixtures containing $Fe(CN)_6^{4-}$ were titrated with NaCl 2 M.

Supplementary Table 1: Critical salt concentration. CSC of $Fe(CN)_6^{3-}$/$Fe(CN)_6^{4-}$-pLys mixtures at different $Fe(CN)_6^{3-}$ / $Fe(CN)_6^{4-}$concentrations, calculated from plots in Supplementary Figure 10, and the corresponding salt concentrations that allow for the regulation of the assembly of these coacervates by redox chemistry.

Lys (mM)	$Fe(CN)_6^{3-}$ (mM)	$CSC_{ferricyanide}$ (mM)	$Fe(CN)_6^{4-}$ (mM)	$CSC_{ferrocyanide}$ (mM)	Salt concentrations allowing for redox regulation
5	1	0	1	330	0-330
5	2	260	2	548	260-548
5	3	330	3	570	330-570
5	4	430	4	595	430-595
5	5	460	5	620	460-620
5	6	460	6	670	460-670

2.6. *Redox reactions inside coacervates*

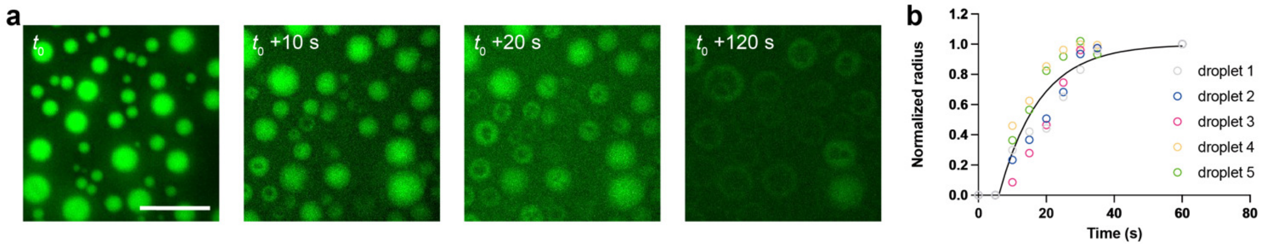

Supplementary Figure 11: NADPH oxidation. (a) Confocal micrographs of $Fe(CN_6)^{3-}$/$(Arg)_{10}$ protocells (stained with NADPH), time series of representative protocells after pH-triggered redox reaction. The experiment was independently repeated three times with similar results. Scale bar, 10 µm. (b) The increase of the radius of the fluorescence depletion zone over time. We analyzed the increase of the radius (average of 5 droplets) over time by fitting to an exponential growth of the form $R = 1 - e^{-(t-t_0)/\tau}$, where τ is the characteristic timescale of diffusion.

3. Peptide bond formation reactions

3.1. Control reactions without ferricyanide

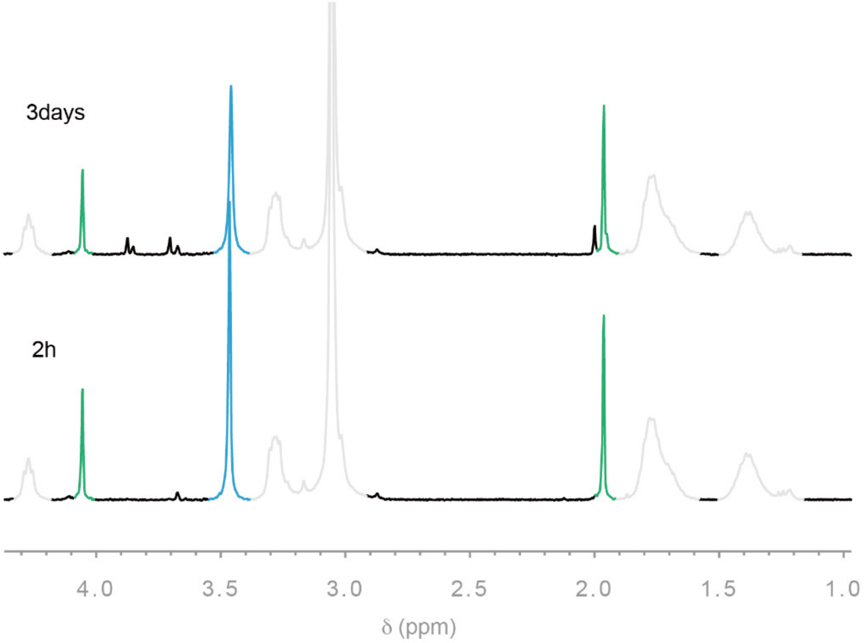

Supplementary Figure 12: Ligation reaction without ferricyanide. ^{1}H NMR spectrum of peptide ligation reaction in control solution without $Fe(CN_6)^{3-}$, no ligation product observed after 3 h. (Ac-Gly-SH (8mM, green), Gly (3 equiv., blue), ($Fe(CN_6)^{3-}$, 0mM), ($(Lys(Me)_3)_{30}$, gray)). The small amount of product formed after 3 days may be due to O_2-mediated oxidation.

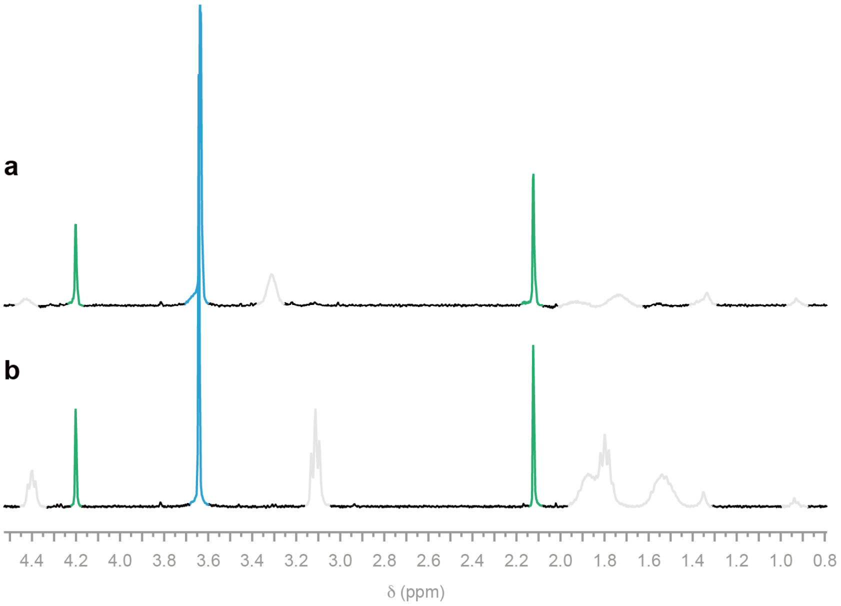

4.

Supplementary Figure 13: Ligation reaction with ferrocyanide. ^{1}H NMR spectrum of peptide ligation reaction in $Fe(CN_6)^{4-}/(Arg)_{10}$ (a) and $Fe(CN_6)^{4-}/(Lys)_{20}$ protocells, no ligation product observed after 3 h. (Ac-Gly-SH (8 mM, green), Gly (3 equiv., blue), ($Fe(CN_6)^{4-}$, 6 mM), (polycations, gray)). Peaks are slightly shifted with respect to Supplementary figure 12, due to the presence of paramagnetic $Fe(CN_6)^{3-}$.

3.2. *Reference ligation reaction in supernatant phase*

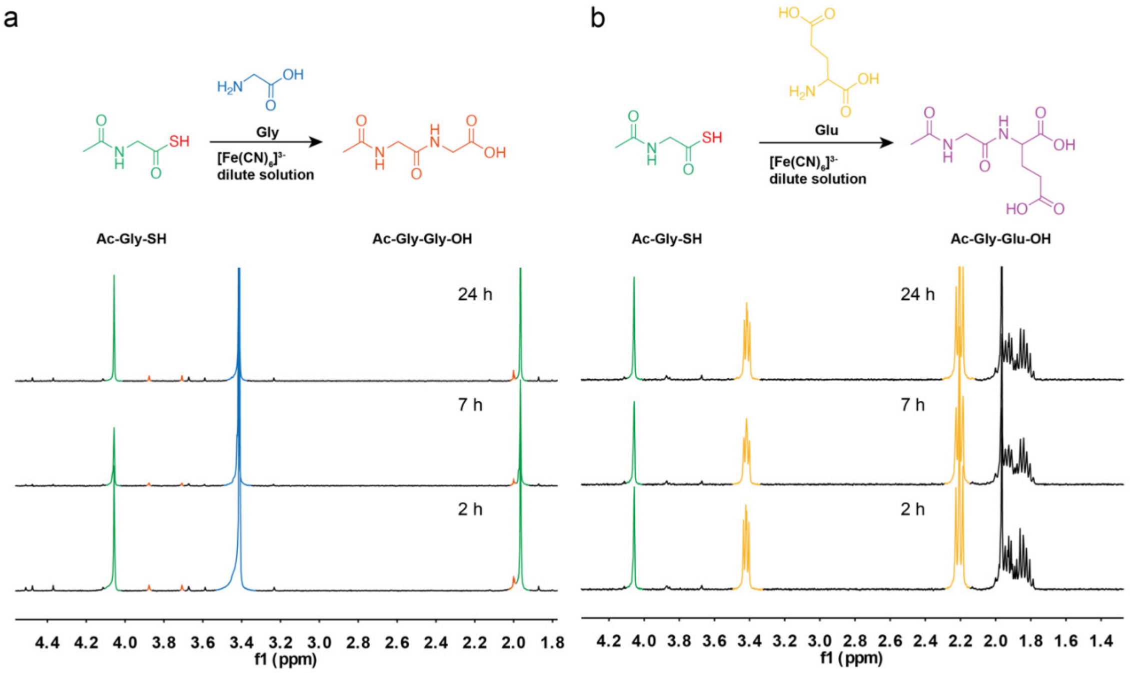

Supplementary Figure 14: Ligation reaction in supernatant. (a) Time series of ^{1}H NMR spectrum of peptide ligation reaction in the dilute phase. (Ac-Gly-SH (8 mM, green), Gly (24 mM, blue), $Fe(CN_6)^{3-}$ (0.8 mM)). (b) As in (a) for peptide ligation reaction with glutamic acid in the dilute phase. (Ac-Gly-SH (8 mM, green), Glu (24 mM, yellow), $Fe(CN_6)^{3-}$ (0.8 mM)).

3.3. *Control reactions with coacervates in borate buffer*

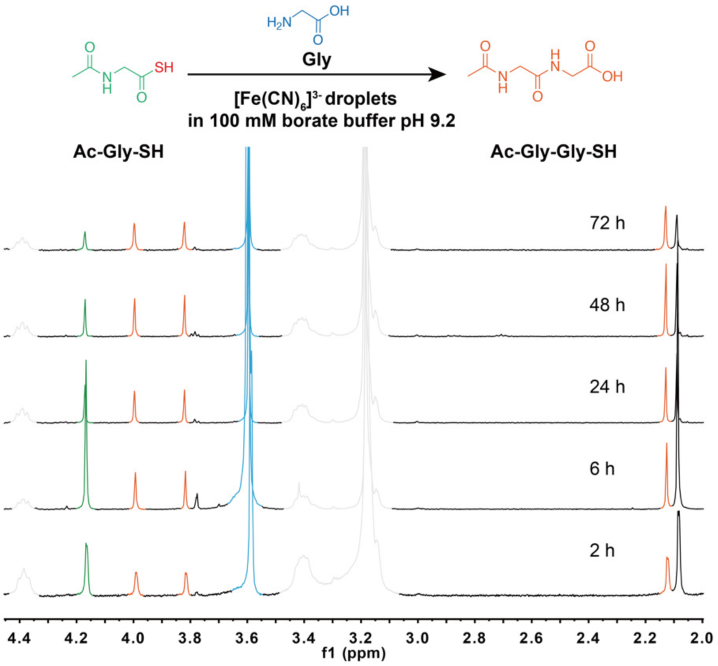

Supplementary Figure 15: Ligation reaction in borate buffer. Time series of ^{1}H NMR spectrum of peptide ligation reaction in $Fe(CN_6)^{3-}/(Lys(Me)_3)_{20}$ protocells in 100 mM borate buffer pH 9.2. (Ac-Gly-SH (8 mM, green), Gly (24 mM, yellow), ($Fe(CN_6)^{3-}$, 8 mM), (polycations, gray)).

3.4. Coacervate-mediated ligation reactions with individual amino acids

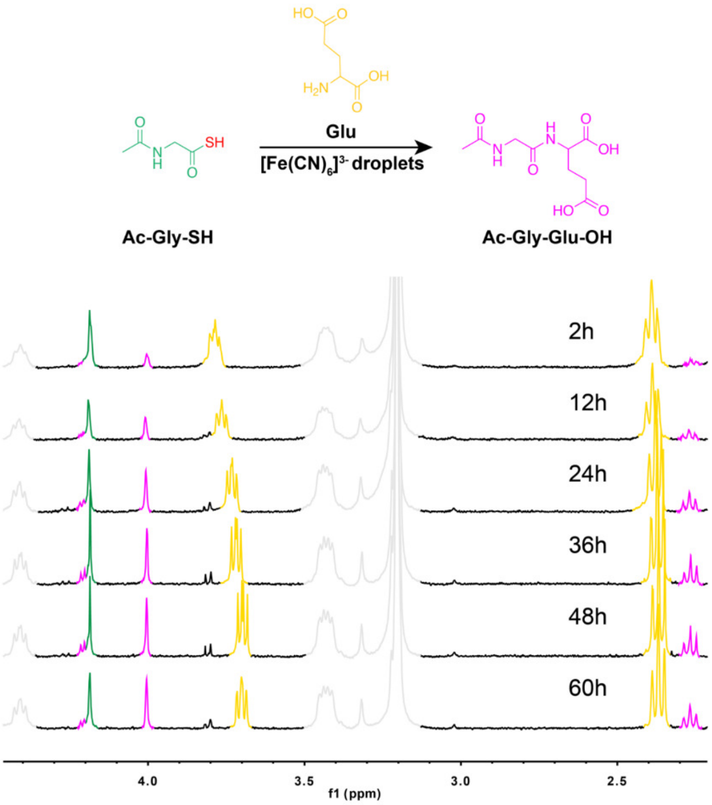

Supplementary Figure 16: Ligation reaction with glutamic acid. ^{1}H NMR time series of peptide ligation reaction in $Fe(CN_6)^{3-}/(Lys(Me)_3)_{20}$ protocells. (Ac-Gly-SH (8 mM, green), Glu (24 mM, yellow), ($Fe(CN_6)^{3-}$, 8 mM), (polycations, gray)).

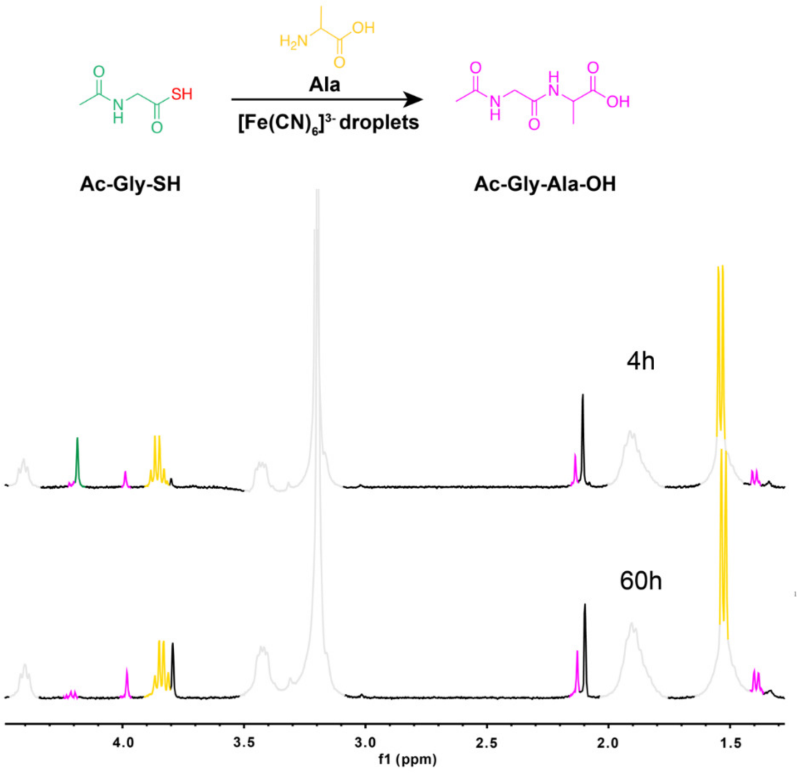

Supplementary Figure 17: Ligation reaction with alanine. ^{1}H NMR time series of peptide ligation reaction in $Fe(CN_6)^{3-}$/$(Lys(Me)_3)_{20}$ protocells. (Ac-Gly-SH (8 mM, green), Ala (24 mM, yellow), ($Fe(CN_6)^{3-}$, 8 mM), (polycations, gray)).

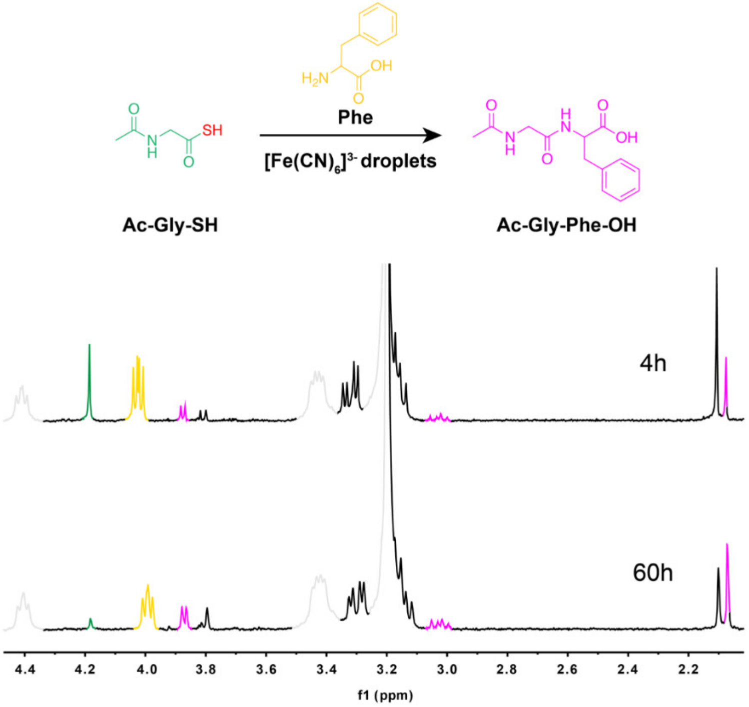

Supplementary Figure 18: Ligation reaction with phenylalanine. ^{1}H NMR time series of peptide ligation reaction in $Fe(CN_6)^{3-}$/$(Lys(Me)_3)_{20}$ protocells. (Ac-Gly-SH (8 mM, green), Phe (24 mM, yellow), ($Fe(CN_6)^{3-}$, 8 mM), (polycations, gray)).

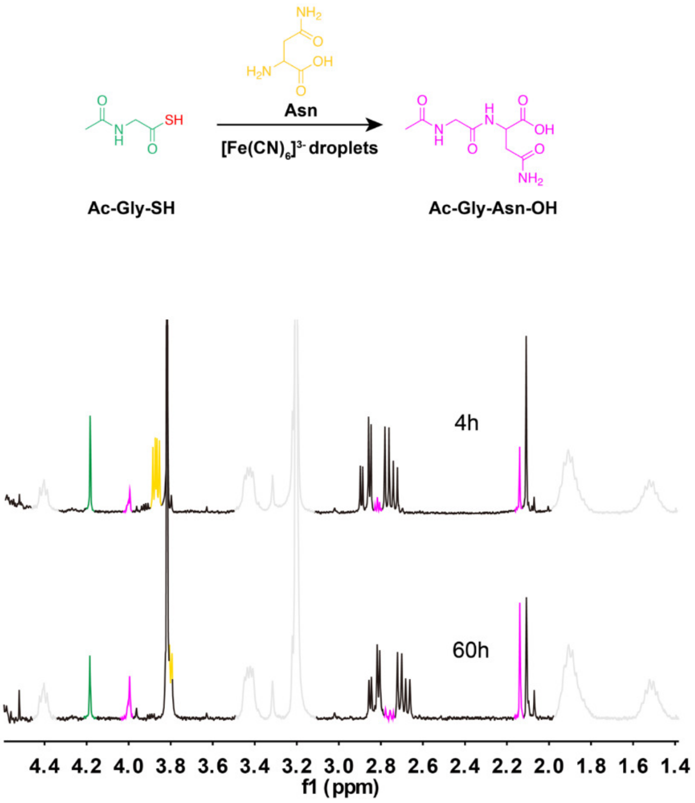

Supplementary Figure 19: Ligation reaction with asparagine. ^{1}H NMR time series of peptide ligation reaction in $Fe(CN_6)^{3-}$/$(Lys(Me)_3)_{20}$ protocells. (Ac-Gly-SH (8 mM, green), Asn (24 mM, yellow), ($Fe(CN_6)^{3-}$, 8 mM), (polycations, gray)).

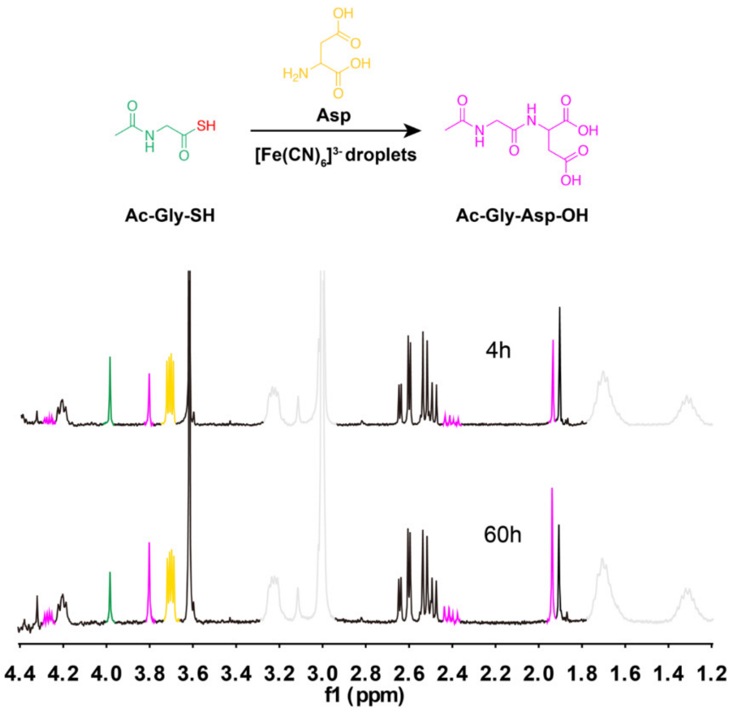

Supplementary Figure 20: Ligation reaction with aspartic acid. ^{1}H NMR time series of peptide ligation reaction in $Fe(CN_6)^{3-}$/$(Lys(Me)_3)_{20}$ protocells. (Ac-Gly-SH (8 mM, green), Asp (24 mM, yellow), ($Fe(CN_6)^{3-}$, 8 mM), (polycations, gray)).

3.5. *Coacervate-mediated ligation reactions with mixtures of amino acids*

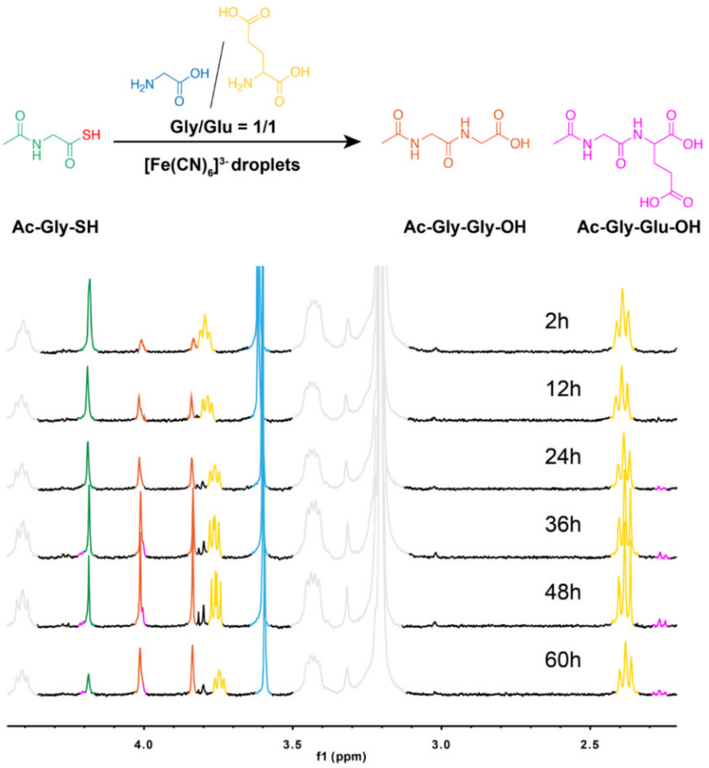

Supplementary Figure 21: Ligation reaction with Gly/Glu. ^{1}H NMR time series of peptide ligation reaction in $Fe(CN_6)^{3-}$/$(Lys(Me)_3)_{20}$ protocells. (Ac-Gly-SH (8 mM, green), Glu (12 mM, yellow), Gly (12 mM, blue), ($Fe(CN_6)^{3-}$, 8mM), (polycations, gray)).

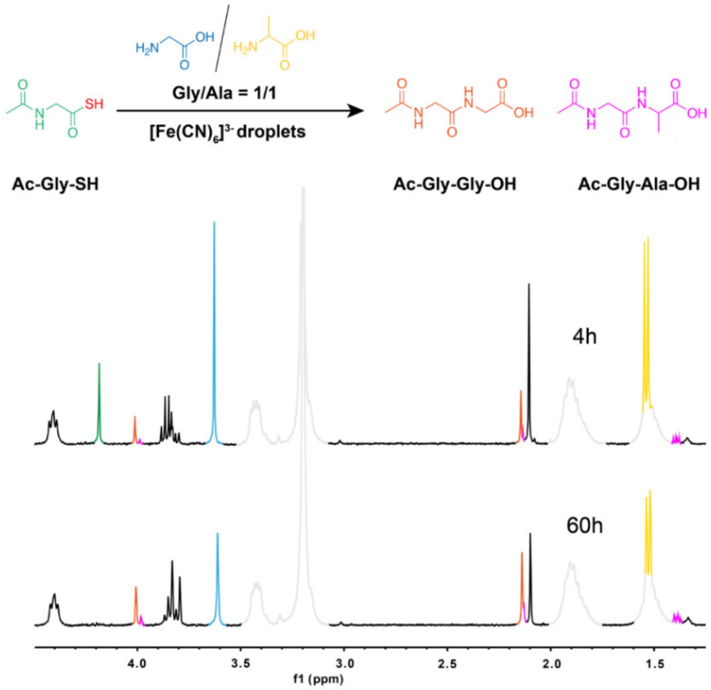

Supplementary Figure 22: Ligation reaction with Gly/Ala. ^{1}H NMR time series of peptide ligation reaction in $Fe(CN_6)^{3-}$/$(Lys(Me)_3)_{20}$ protocells. (Ac-Gly-SH (8 mM, green), Ala (12 mM, yellow), Gly (12 mM, blue), ($Fe(CN_6)^{3-}$, 8mM), (polycations, gray)).

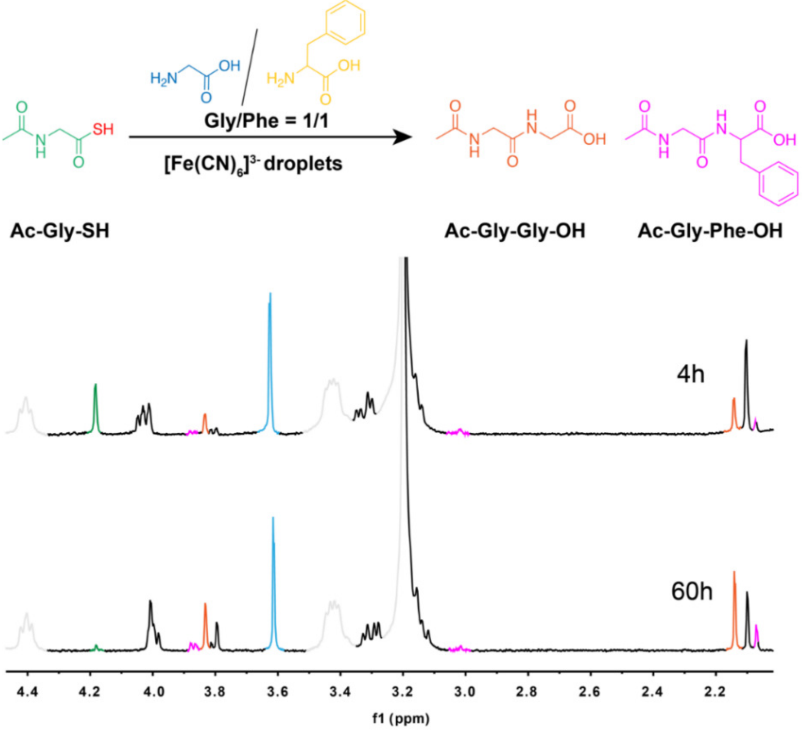

Supplementary Figure 23: Ligation reaction with Gly/Phe. ^{1}H NMR time series of peptide ligation reaction in $Fe(CN_6)^{3-}$e/$(Lys(Me)_3)_{20}$ protocells. (Ac-Gly-SH (8 mM, green), Phe (12 mM, yellow), Gly (12 mM, blue), ($Fe(CN_6)^{3-}$, 8mM), (polycations, gray)).

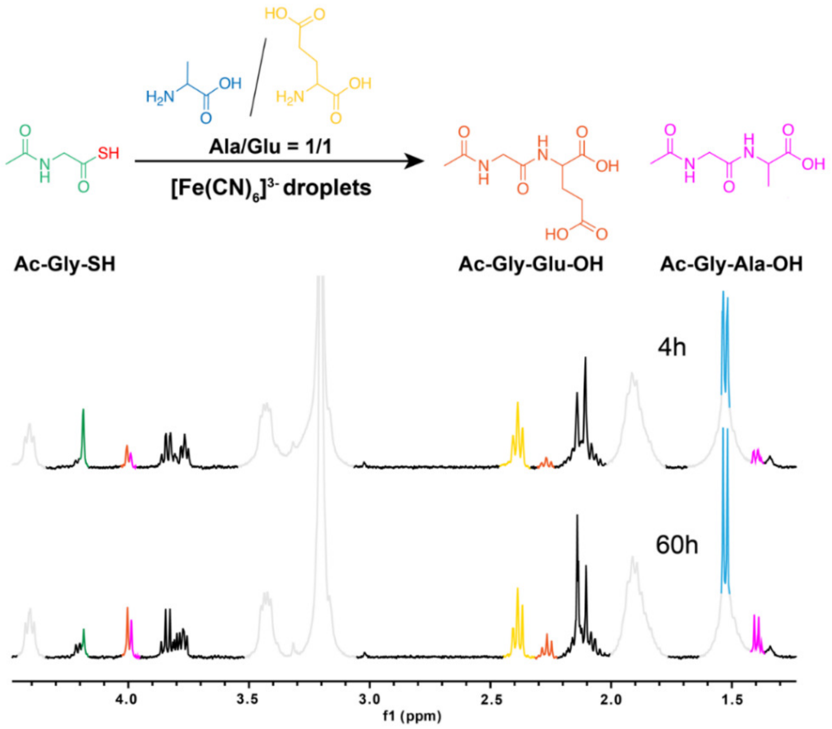

Supplementary Figure 24: Ligation reaction with Ala/Glu. ^{1}H NMR time series of peptide ligation reaction in $Fe(CN_6)^{3-}$/$(Lys(Me)_3)_{20}$ protocells. (Ac-Gly-SH (8 mM, green), Ala (12 mM, yellow), Glu (12 mM, blue), ($Fe(CN_6)^{3-}$, 8mM), (polycations, gray)).

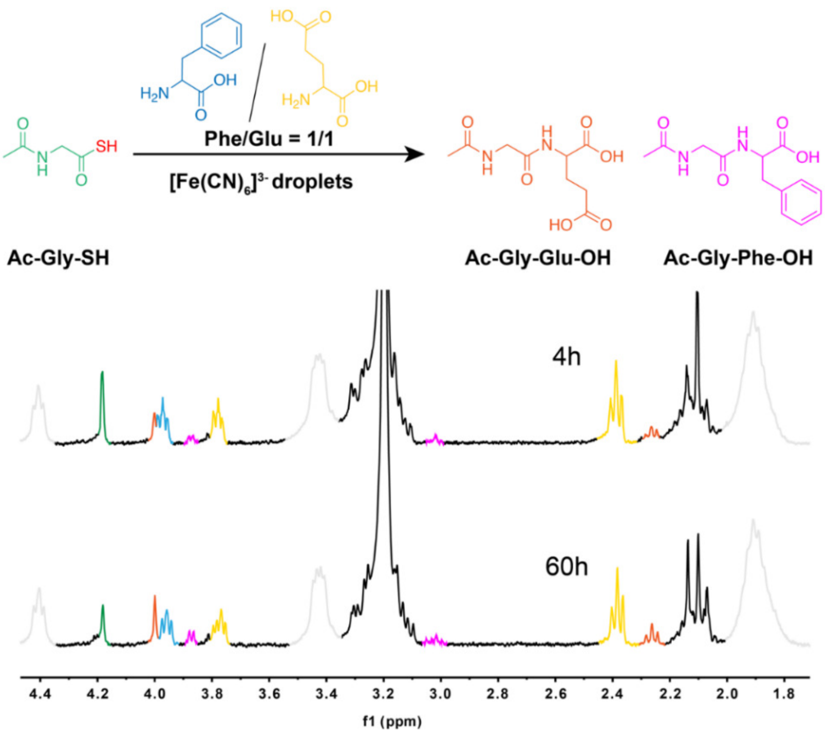

Supplementary Figure 25: Ligation reaction with Phe/Glu. ^{1}H NMR time series of peptide ligation reaction in $Fe(CN_6)^{3-}$/$(Lys(Me)_3)_{20}$ protocells. (Ac-Gly-SH (8 mM, green), Phe (12 mM, yellow), Glu (12 mM, blue), ($Fe(CN_6)^{3-}$, 8mM), (polycations, gray)).

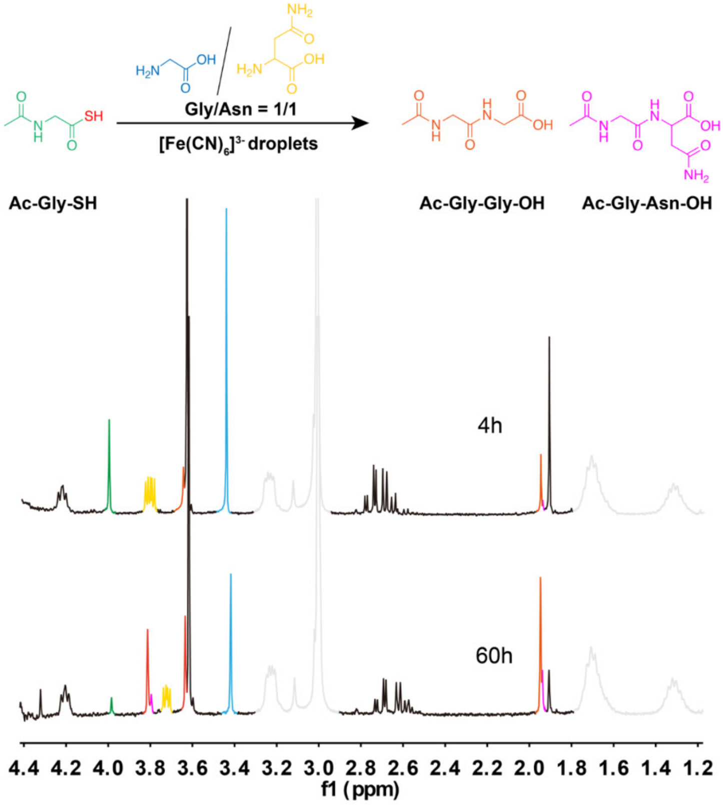

Supplementary Figure 26: Ligation reaction with Gly/Asn. ^{1}H NMR time series of peptide ligation reaction in $Fe(CN_6)^{3-}$/$(Lys(Me)_3)_{20}$ protocells. (Ac-Gly-SH (8 mM, green), Asn (12 mM, yellow), Gly (12 mM, blue), $Fe(CN_6)^{3-}$, 8 mM).

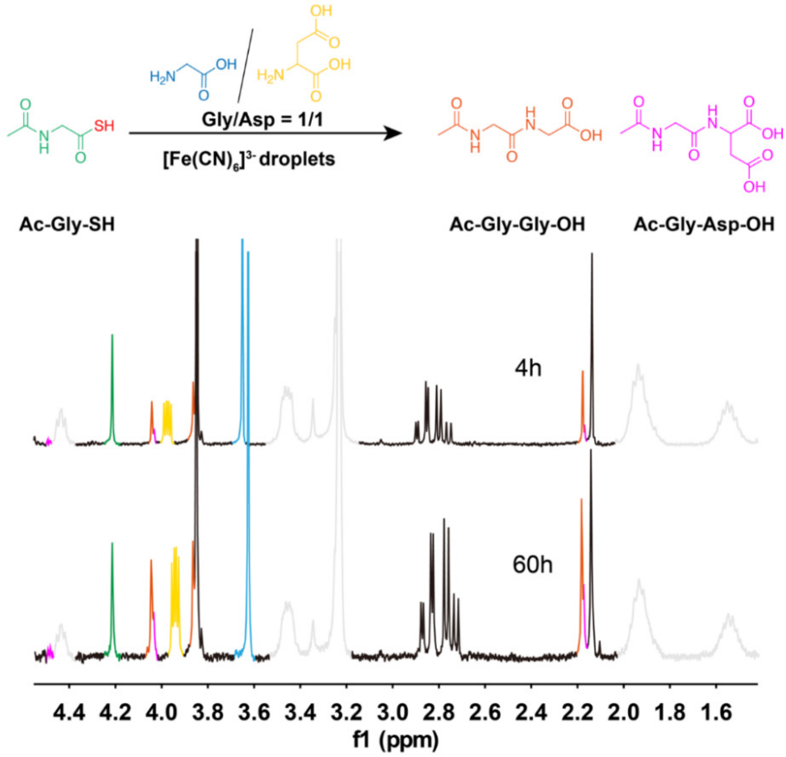

Supplementary Figure 27: Ligation reaction with Gly/Asp. ^{1}H NMR time series of peptide ligation reaction in $Fe(CN_6)^{3-}$/$(Lys(Me)_3)_{20}$ protocells. (Ac-Gly-SH (8 mM, green), Asp (12 mM, yellow), Gly (12 mM, blue), $Fe(CN_6)^{3-}$, 8 mM).

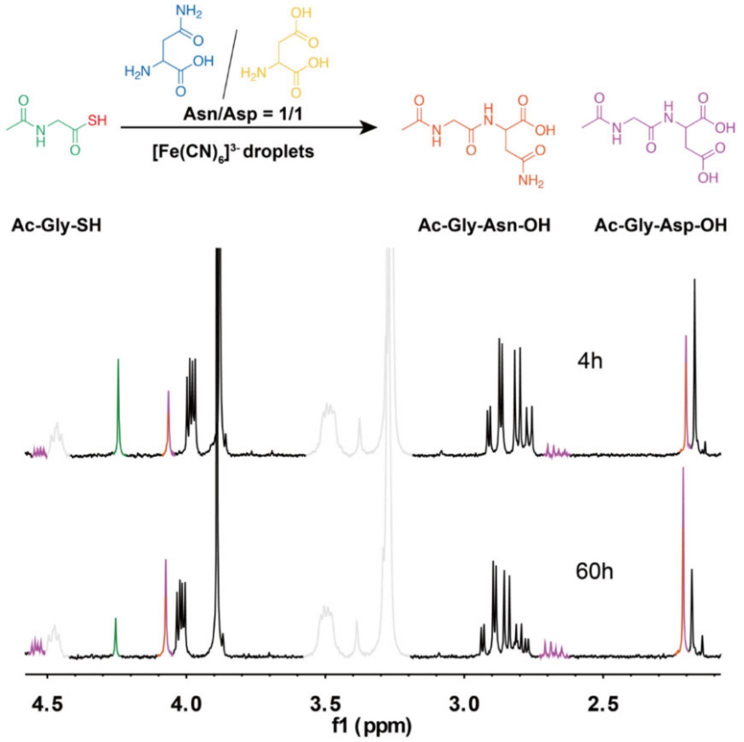

Supplementary Figure 28: Ligation reaction with Asn/Asp. ^{1}H NMR time series of peptide ligation reaction in $Fe(CN_6)^{3-}$/$(Lys(Me)_3)_{20}$ protocells. (Ac-Gly-SH (8 mM, green), Asp (12 mM, yellow), Asn (12 mM, blue), ($Fe(CN_6)^{3-}$, 8mM), (polycations, gray)).

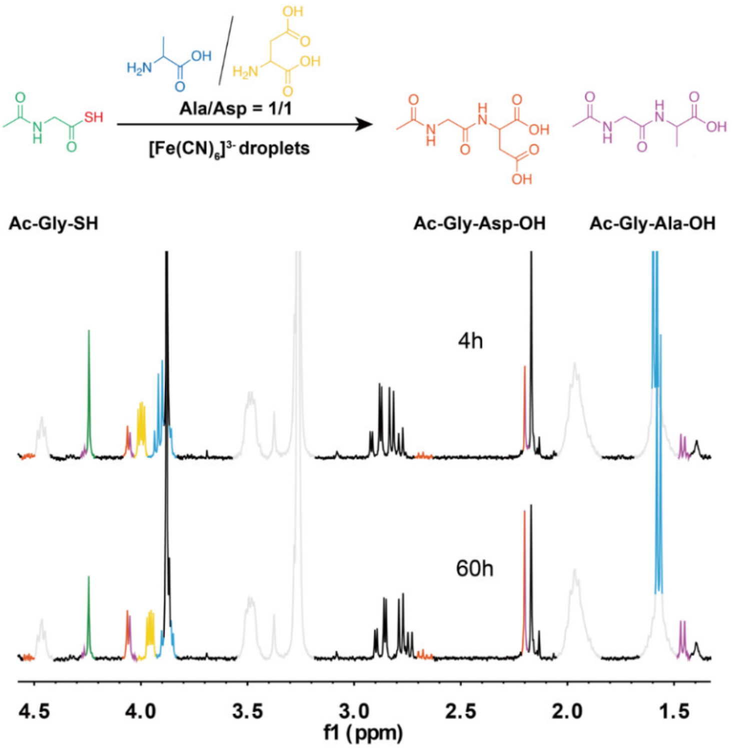

Supplementary Figure 29: Ligation reaction with Ala/Asp. ^{1}H NMR time series of peptide ligation reaction in $Fe(CN_6)^{3-}$ /$(Lys(Me)_3)_{20}$ protocells. (Ac-Gly-SH (8 mM, green), Asp (12 mM, yellow), Ala (12 mM, blue), ($Fe(CN_6)^{3-}$, 8mM), (polycations, gray)).

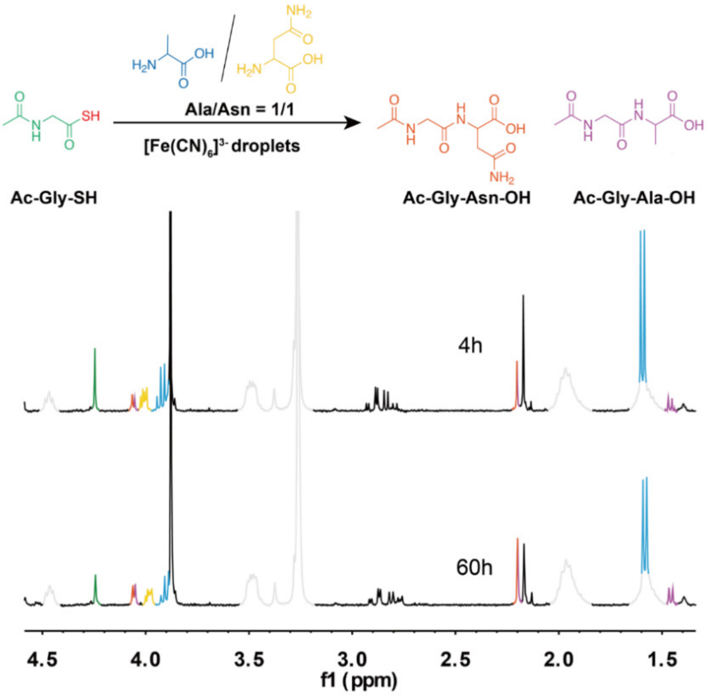

Supplementary Figure 30: Ligation reaction with Ala/Asn. ^{1}H NMR time series of peptide ligation reaction in $Fe(CN_6)^{3-}$ /$(Lys(Me)_3)_{20}$ protocells. (Ac-Gly-SH (8 mM, green), Asn (12 mM, yellow), Ala (12 mM, blue), ($Fe(CN_6)^{3-}$, 8mM), (polycations, gray)).

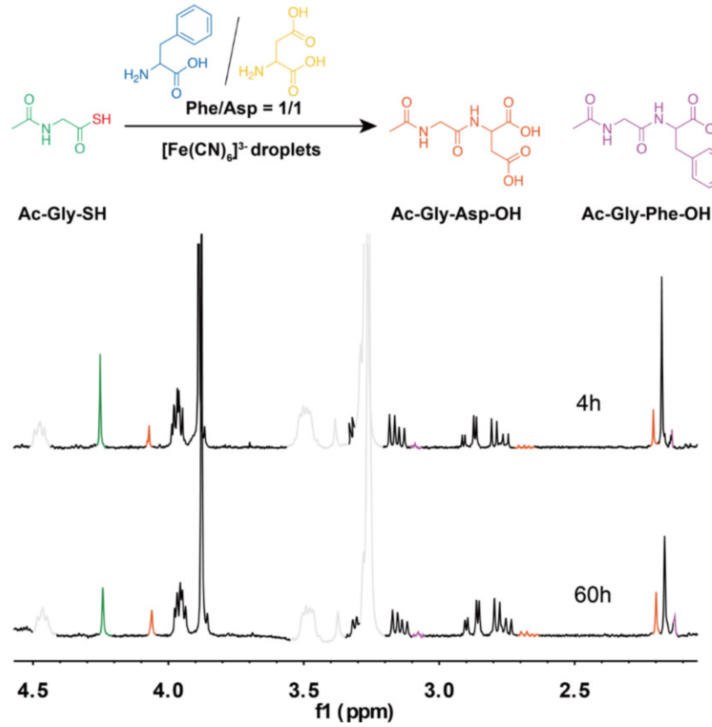

Supplementary Figure 31: Ligation reaction with Phe/Asp. ^{1}H NMR time series of peptide ligation reaction in $Fe(CN_6)^{3-}$/$(Lys(Me)_3)_{20}$ protocells. (Ac-Gly-SH (8 mM, green), Asp (12 mM, yellow), Phe (12 mM, blue), ($Fe(CN_6)^{3-}$, 8mM), (polycations, gray)).

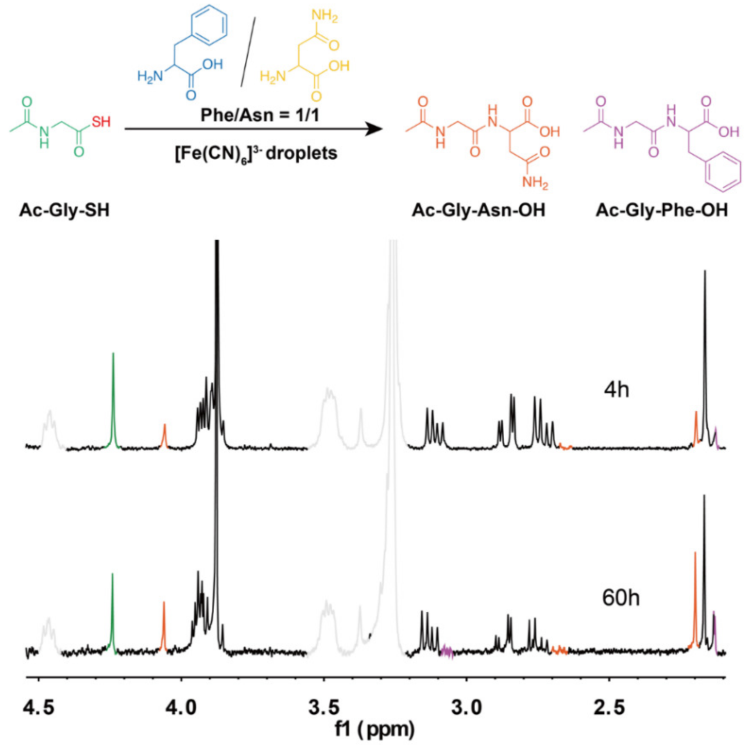

Supplementary Figure 32: Ligation reaction with Phe/Asn. ^{1}H NMR time series of peptide ligation reaction in $Fe(CN_6)^{3-}$/$(Lys(Me)_3)_{20}$ protocells. (Ac-Gly-SH (8 mM, green), Asn (12 mM, yellow), Phe (12 mM, blue), ($Fe(CN_6)^{3-}$, 8mM), (polycations, gray)).

4. Fiber self-assembly inside coacervates

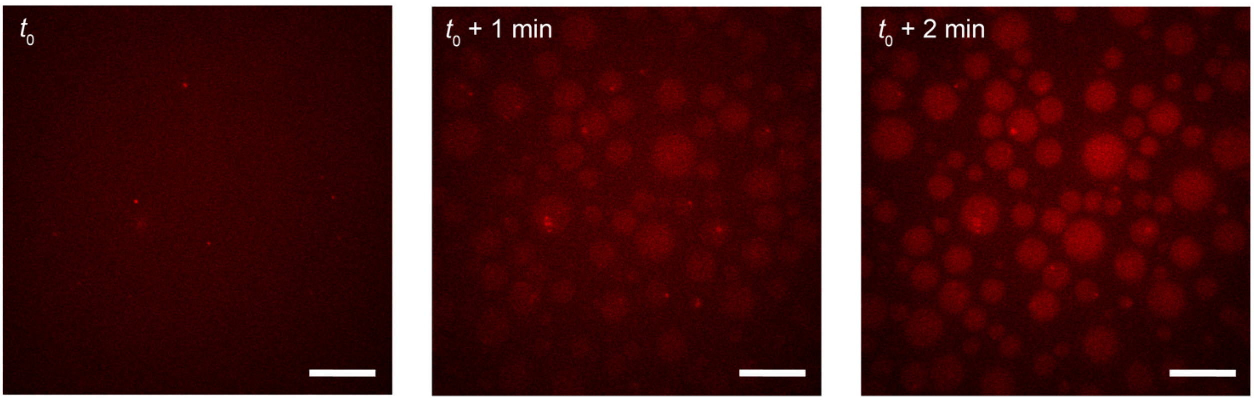

Supplementary Figure 33: Fiber assembly in oligoarginine coacervates. Time-dependent CLSM fluorescence images of $Fe(CN_6)^{3-}/(Arg)_{10}$ droplets showing increase in red fluorescence throughout the coacervate interior of the protocell over a period of 2 min. The reaction product negatively charged DBC^{2-} accumulates in the molecularly crowded coacervate interior. The experiment was independently repeated three times with similar results. Scale bar, 10 µm.

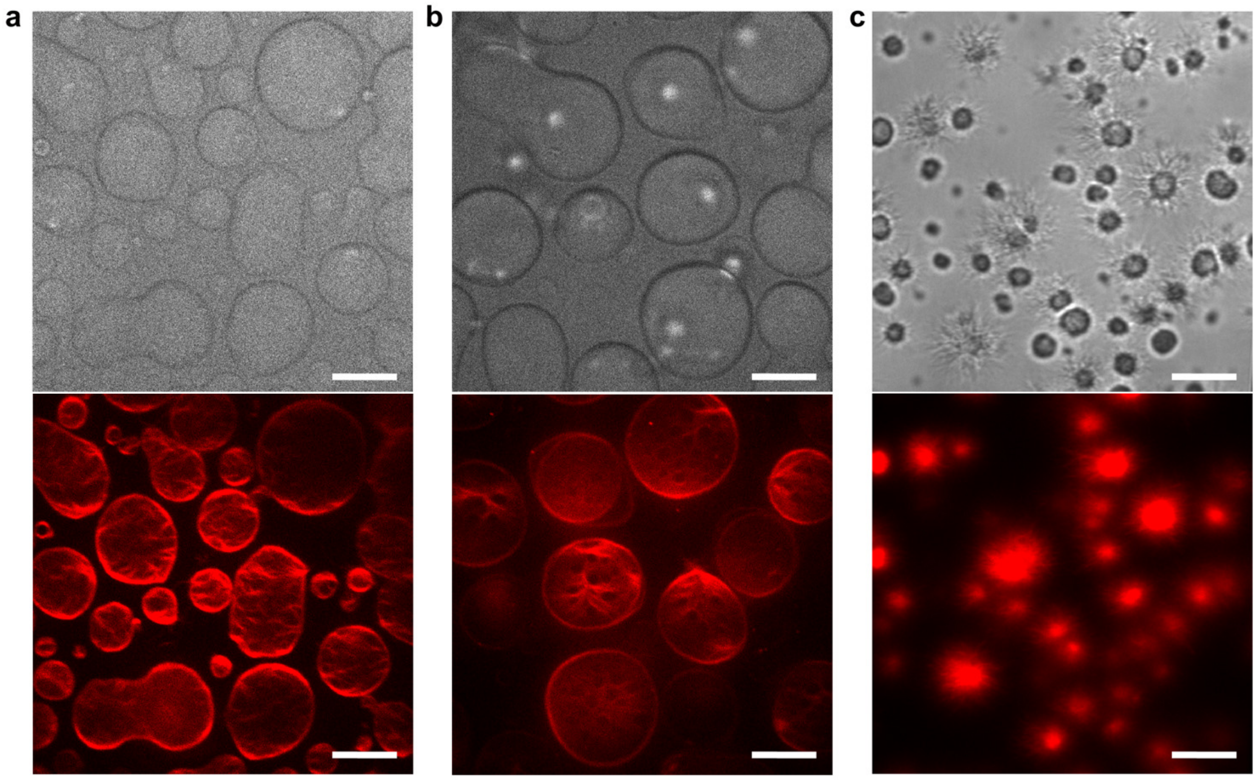

Supplementary Figure 34: Fiber assembly in oligolysine and methylated oligolysine coacervates. CLSM fluorescence images of $Fe(CN_6)^{3-}/(Lys(Me)_3)_{20}$ (a), $Fe(CN_6)^{3-}/(Lys(Me)_3)_{30}$ (b), and $Fe(CN_6)^{3-}/(Lys)_{30}$ (c) protocells (with dye, Nile red) after addition of BC (pH = 3). The experiments in **a-c** were independently repeated three times with similar results. Scale bar, 10 µm.

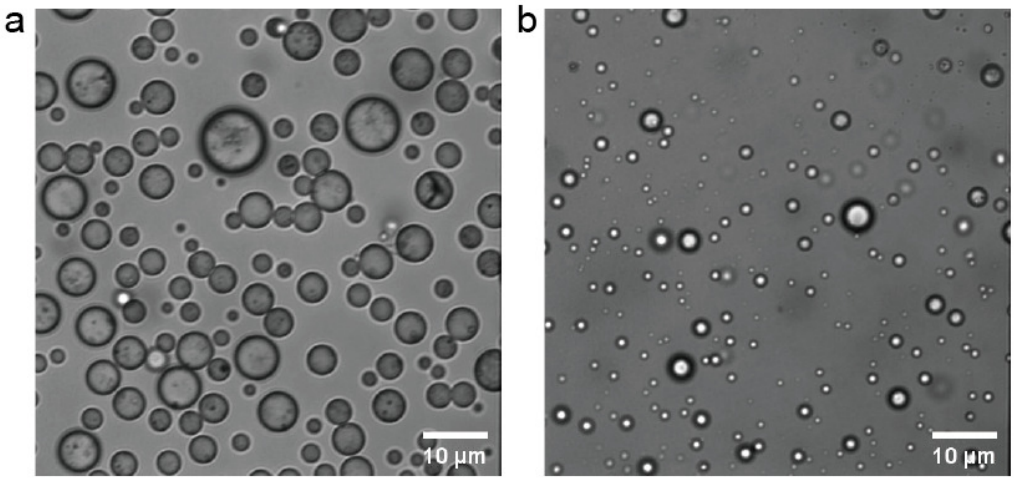

Supplementary Figure 35: Dissolution of fibers in coacervates by base. (a) Confocal micrographs of $Fe(CN)_6^{3-}/(Arg)_{10}$ protocells, with addition of BC^- (final concentration $Fe(CN_6)^{3-}$ 4 mM, $(Arg)_{10}$ 12 mM, BC^- 10 mM) in pH 3.0 by adding 1 µL 1 M HCl. (b) Image shows the fibers inside the droplets dissolved by adding 2 µL 1 M NaOH to increase the pH to 7.0. The experiments in **a**-**b** were independently repeated three times with similar results. Scale bar, 10 µm.

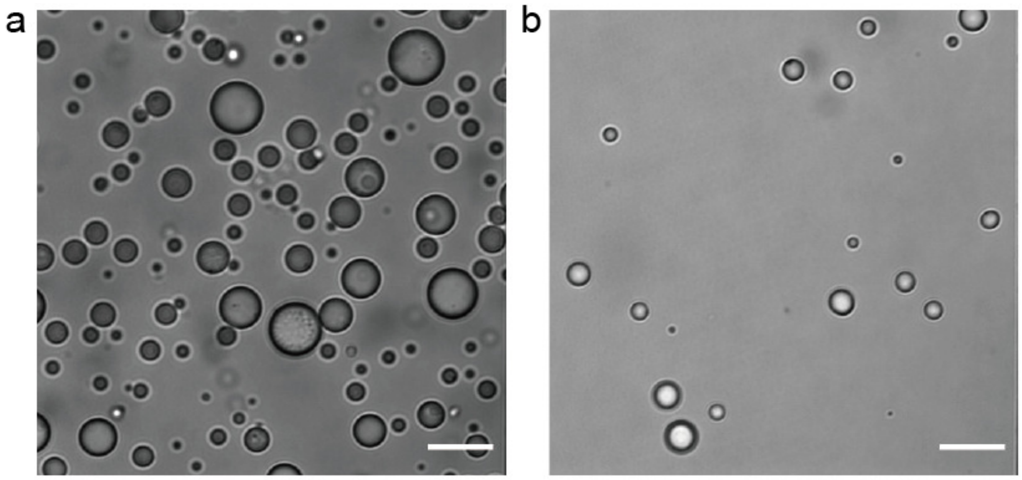

Supplementary Figure 36: Dissolution of fibers in coacervates by reduction. (a) Confocal micrograph of $Fe(CN)_6^{3-}/(Arg)_{10}$ protocells, with addition of BC^- (final concentration $Fe(CN_6)^{3-}$ 4 mM, $(Arg)_{10}$ 12 mM, BC^- 10 mM) in pH 3.0 by adding 1 µL 1 M HCl. (b) Image shows the fibers inside the droplets dissolved by adding 5 µL 50 mM β-mercaptoethanol. The experiments in **a**-**b** were independently repeated three times with similar results. Scale bar, 10 µm.

5. Reaction with aminonitriles

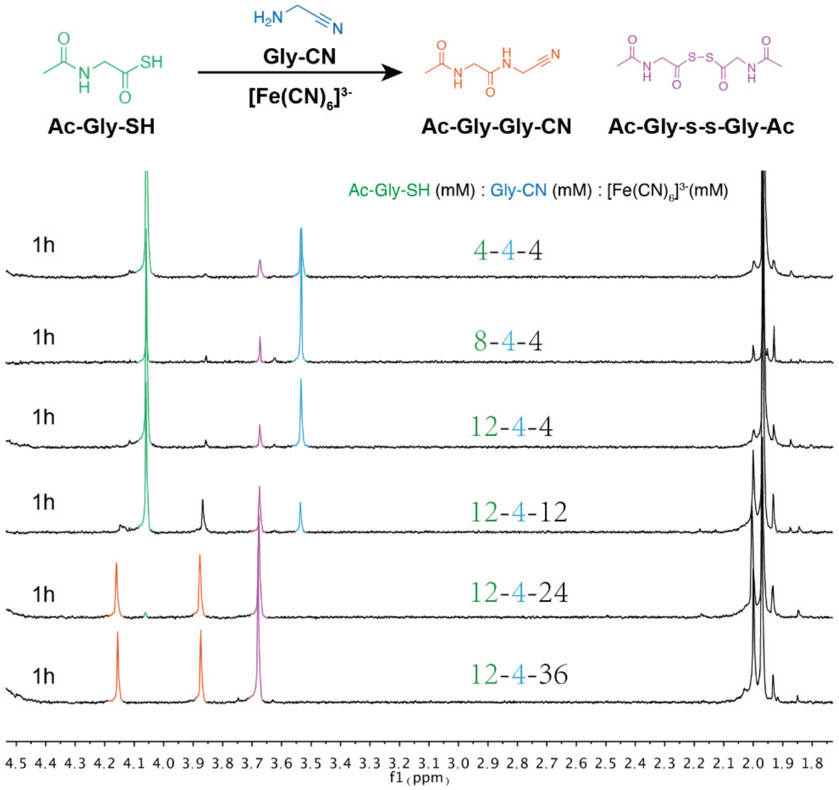

Supplementary Figure 37: Ligation reaction with Gly-CN. ^{1}H NMR spectrum of peptide ligation reaction with Gly-CN in $Fe(CN_6)^{3-}$ solution.

Steps	Step 1	Step 2	Step 3	Step 4	Step 5	Step 6	Step...
Components	D_2O	pLys(Me)$_3$	ferricyanide	AA or AA$_1$:AA$_2$ 1:1	1 M NaOD	Ac-Gly-SH	1 M NaOD
Volume added	128 μl	24 μl	8 μl	24 μl or 12 μl : 12 μl	0.2 μl*n	16 μl	0.2 μl*m
Final (mM)		24 mM	8 mM	24 mM or 12 mM : 12 mM	pH = 9	8 mM	pH = 9

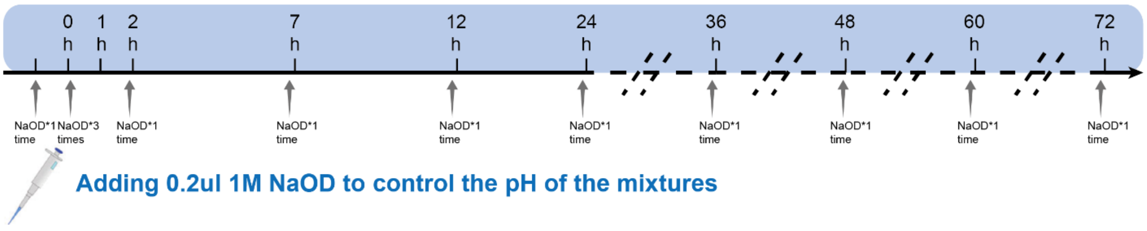

Supplementary Figure 38: Addition order and base addition during ligation reaction. Addition order of reactants and coacervate components in peptide ligation reaction with coacervates (top), and typical addition sequence of aliquots of base to maintain a constant pH around 9 during peptide ligation reaction.

6. Supplementary references

1. Granados, E. N.; Bello, J., Alkylated poly(amino acids). I. Conformational properties of poly(Nε-trimethyl- L-lysine) and poly(Nδ-trimethyl-L-ornithine). *Biopolymers* **1976,** *18*, 1479-1486.
2. Okamoto, R.; Haraguchi, T.; Nomura, K.; Maki, Y.; Izumi, M.; Kajihara, Y., Regioselective alpha-Peptide Bond Formation Through the Oxidation of Amino Thioacids. *Biochemistry* **2019,** *58* (12), 1672-1678.
3. Canavelli, P.; Islam, S.; Powner, M. W., Peptide ligation by chemoselective aminonitrile coupling in water. *Nature* **2019,** *571* (7766), 546-549.
4. Harpaz, Y.; Gerstein, M.; Chothia, C., Volume changes on protein folding. *Structure* **1994,** *2*, 641.
